# HIV treatment for prevention

**DOI:** 10.1186/1758-2652-14-28

**Published:** 2011-05-25

**Authors:** Juan Ambrosioni, Alexandra Calmy, Bernard Hirschel

**Affiliations:** 1 HIV Unit, Department of Infectious Diseases, University of Geneva Hospitals, Geneva, Switzerland; 2 MSF Access Campaign to Essential Medicine, Geneva, Switzerland

## Abstract

"No virus, no transmission." Studies have repeatedly shown that viral load (the quantity of virus present in blood and sexual secretions) is the strongest predictor of HIV transmission during unprotected sex or transmission from infected mother to child.

Effective treatment lowers viral load to undetectable levels. If one could identify and treat all HIV-infected people immediately after infection, the HIV/AIDS epidemic would eventually disappear.

Such a radical solution is currently unrealistic. In reality, not all people get tested, especially when they fear stigma and discrimination. Thus, not all HIV-infected individuals are known. Of those HIV-positive individuals for whom the diagnosis *is *known, not all of them have access to therapy, agree to be treated, or are taking therapy effectively. Some on effective treatment will stop, and in others, the development of resistance will lead to treatment failure. Furthermore, resources are limited: should we provide drugs to asymptomatic HIV-infected individuals without indication for treatment according to guidelines in order to prevent HIV transmission at the risk of diverting funding from sick patients in urgent need?

In fact, the preventive potential of anti-HIV drugs is unknown. Modellers have tried to fill the gap, but models differ depending on assumptions that are strongly debated. Further, indications for antiretroviral treatments expand; in places like Vancouver and San Francisco, the majority of HIV-positive individuals are now under treatment, and the incidence of new HIV infections has recently fallen. However, correlation does not necessarily imply causation. Finally, studies in couples where one partner is HIV-infected also appear to show that treatment reduces the risk of transmission.

More definite studies, where a number of communities are randomized to either receive the "test-and-treat" approach or continue as before, are now in evaluation by funding agencies. Repeated waves of testing would precisely measure the incidence of HIV infection. Such trials face formidable logistical, practical and ethical obstacles. However, without definitive data, the intuitive appeal of "test-and-treat" is unlikely to translate into action on a global scale. In the meantime, based on the available evidence, we must strive to provide treatment to all those in medical need under the current medical guidelines. This will lead to a decrease in HIV transmission while "test-and-treat" is fully explored in prospective clinical trials.

## Review

Approximately 2.7 million new HIV infections were diagnosed in 2009, mostly in resource-poor countries. In 2009, the estimated number of new HIV infections was approximately 21% lower than at the epidemic's peak 12 years earlier. As a result of the combined effect of increased longevity of treated patients and decrease of new infections, prevalence is stabilizing [1].

Highly active antiretroviral therapy (HAART) has substantially reduced AIDS-related hospital admissions and death rates [2-5]. In the past decade, HAART has become simpler, better tolerated, less toxic, more effective and less expensive. There is evidence for an increase in complications in untreated individuals, even at relatively high CD4 T cell counts [6,7]. As a consequence, indications for HAART have expanded to include most HIV-infected patients regardless of their CD4 cell counts [8-10].

In contrast to the proven individual benefits of HAART, its preventive role has been analyzed less. Only in recent years has the expansion of HAART been considered as a tool to limit the growth of the HIV epidemic [11], in addition to the classical preventive measures, such as condoms or clean needles for injecting drug users (IDUs). The viral load correlates with the rate of heterosexual [12] and mother to child transmission [13]. Since HAART decreases viral load in the blood and in genital secretions[14,15], it potentially reduces the risk of HIV transmission.

This article represents the authors' view, expressed in a plenary talk given at the XVIII International AIDS Conference (AIDS 2010) in Vienna, Austria. The most important evidence regarding the preventive effect of the treatment of HIV infection is highlighted, without attempting to perform a comprehensive review.

### Most relevant available evidence

#### Mother to child transmission

Although the biology of HIV sexual transmission is very different to that of mother to child transmission (MTCT), the use of antiretroviral drugs in this setting as a preventive tool was one of the first and more powerful pieces of evidence for the effectiveness of this strategy. In the ACTG076 trial published in 1994, zidovudine was administered to the mother during pregnancy and labour, and to the newborn during the first four weeks of life. MTCT fell from 25.5% to 8.3% [16].

Since then, using antiretrovirals (ARVs) has reduced transmission to less than 1% [13]. ARVs reduce MTCT mainly by decreasing maternal viral load in the blood and genital secretions. An additional mechanism of protection is infant prophylaxis, because antiretroviral drugs cross the placenta. This results in adequate systemic drug levels in the infant during the passage through the birth canal, a time of intensive exposure to HIV [17]. In developed countries, treatment with HAART is now the standard of care in all HIV-positive pregnant women, given its high effectiveness in preventing MTCT.

#### Studies in serodiscordant couples

Heterosexual transmission is the main route of HIV spread worldwide. Coinciding with ACTG 076, Musicco *et al *published the results of zidovudine monotherapy in preventing HIV transmission among 436 serodiscordant couples where the male partner was HIV positive. The rate of transmission from zidovudine-treated men was lower than from untreated men (relative risk, 0.5; 95% confidence interval, 0.1 to 0.9) [18]. More recently, Donnell and colleagues [19] compared the HIV incidence rate before and after the initiation of HAART in such couples. HIV incidence was 2.24 per 100 person-years before starting HAART (102 transmission events) and 0.37 per 100 person-years after starting HAART (single transmission event), representing a 12-fold reduction in transmission. As expected, most of the transmission events occurred in couples in which the index case had a high viral load.

In another study from Spain [20], 476 serodiscordant heterosexual couples were recruited between 1989 and 2008. The only risk factor for the HIV-negative member of the couple was exposure to the HIV-infected partner. At enrolment, when the index member was on HAART, none of the partners were infected, compared with 9.2% of the partners of untreated patients. During the follow-up period, there was no HIV transmission in couples where the index case was on HAART.

Although these studies provide valuable information, they should not be overestimated. Most of them have serious limitations. For example, it is not possible to ensure that couples are totally monogamous and thus not potentially exposed to other sources of HIV acquisition. Outside the context of serodiscordant couples and MTCT, little information is available on the preventive effect of HAART. In particular, controlled studies in men having sex with men and in intravenous drug users are not available, but the introduction and recent expansion of HAART provides opportunities for counting new HIV infections before and after HAART (so-called "ecological" studies) in all transmission groups.

#### Ecological studies

**Figure 1:**
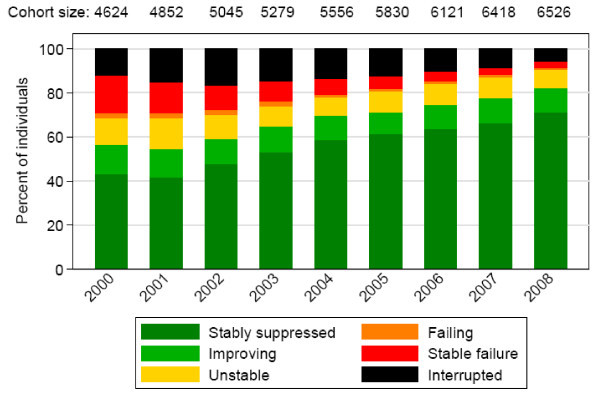
**Viral load categories for participants of the SHCS in Switzerland**. Adapted from Ledergerber *et al*, presented at CROI 2010 (with permission). Viral load categories: Stably suppressed: Three consecutive HIV-1 RNA values below detection limit (<50 copies/mL). Improving: A detectable followed by two undetectable values. Unstable: a) Detectable - undetectable - detectable; or b) Undetectable - detectable - undetectable. Failing: An undetectable followed by two detectable values. Stable failure: Three consecutive detectable viral load values

Although a cause-effect relationship is difficult to prove, ecological studies have correlated the expansion of HAART in a given population and the number of new HIV diagnoses. The most relevant have been performed in high-income countries, where HAART has not only expanded, but has also become more effective. In a recent study from Switzerland performed with participants of the Swiss HIV Cohort Study, the proportion of patients with undetectable viral load progressively increased (Figure 1). Theoretically, as a consequence of HAART expansion and of the increased effectiveness of treatment, the total amount of virus in a given population ("the community viral load") decreases, as does the number of potentially infective patients.

**Figure 2:**
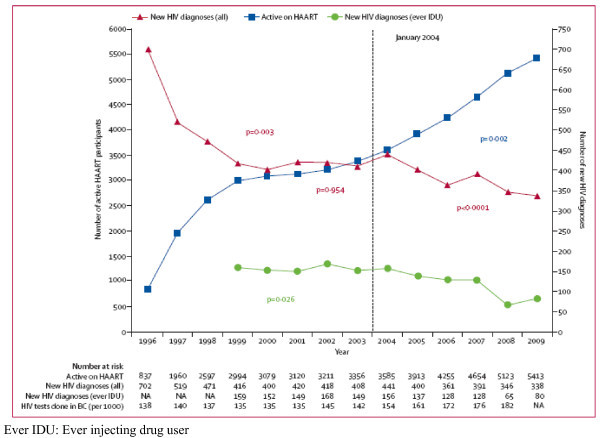
**Number of patients active on HAART and newly discovered HIV in Bristish Columbia, Canada**. Adapted from Montaner *et al*, *Lancet *2010,376(9740):532-539 (with permission).

In British Columbia, Canada, Montaner and colleagues showed an inverse correlation between the expansion of HAART and the number of newly discovered HIV infections [21] (Figure 2). A significant increase in the number of patients on HAART was observed between 1996 and 1999, and this was associated with a reciprocal decrease in the number of new diagnoses. Between 1999 and 2003, the number of treated patients stayed relatively stable, and the same was true of new HIV diagnoses.

Although the biology of blood-borne transmission and sexual transmission differs, the correlation of the community viral load and the HIV transmission rate was particularly suggestive in a cohort of IDUs [22], where all the HIV-negative participants of the cohort were screened for HIV every six months. The average viral load was determined in the HIV-positive participants of the cohort. The authors found a statistically significant correlation between the reduction of the average viral load and the HIV incidence rate. However, it should be noted that this correlation was especially marked in the 1990s following the rapid initial expansion of HAART and less marked more recently.

In San Francisco, where the HIV epidemic is driven mostly by men who have sex with men (MSM), Das Douglas and colleagues also studied the correlation of the "community viral load" and new HIV diagnoses[23]. The mean community viral load declined between 2004 and 2008 secondary to HAART expansion, and correlated with a parallel decrease in the number of new diagnoses (from 798 in 2004 to 434 in 2008). A trend was also found between community viral load and HIV incidence, but this correlation was not statistically significant.

Cowan *et al *showed that in Denmark, more than 80% of infected MSM are on HAART, and more than 85% of these have an undetectable viral load [24]. Despite an increase of unprotected anal intercourse in the MSM population, the number of newly discovered HIV infections remained stable in this group, suggesting a lower rate of transmission per unprotected sex act. This decrease in transmission could be explained by the high HAART coverage of the MSM population, with the undiagnosed or untreated individuals being responsible for most of the new transmission events [24].

In many of these locations, the decrease in HIV transmission coincided with an increase in other sexually transmitted infections (STIs), particularly syphilis [21,23]. STI incidence is an indirect indicator of unsafe sex practices; in addition, ulcerative STIs favour the transmission of HIV. This apparent contradiction - an increase in unsafe sex and in the number of potentially infectious HIV positive individuals due to improved survival, contrasting with a decreasing HIV incidence - is easily resolved when we assume that HIV treatment reduces infectivity for HIV but not, of course, for syphilis and gonorrhoea.

Most of the ecological studies have analyzed the correlation between the proportion of HIV-infected people treated with HAART, and the number of newly diagnosed HIV infections (new HIV diagnoses). However, new HIV diagnoses do not necessarily equal new (or recent) HIV infections: many of these new diagnoses may represent older infections. To better correlate the reduction in the community viral load and HIV transmission, recent infections should be counted, but information on acquisition of infection is usually lacking. This situation could be improved through the widespread use of laboratory tools, such as the quantitative detection of HIV-RNA in seronegative patients, the use of "de-tuned" antibody tests (where antibodies of lower avidity as typically present in recent infections are not recorded as "positive" [25]), or through the analysis of band patterns of immunoblots [26].

#### Mathematical models

A clinical trial providing definitive evidence of the preventive role of antiretroviral therapy in HIV transmission faces numerous obstacles that will be discussed later in this article. It is much cheaper and faster to model the preventive effects of potential interventions, such as increase in HAART coverage at different levels. However, the results of mathematical models depend on the assumptions taken and these assumptions differ greatly between modellers. A complete enumeration or analysis of the numerous models published for HIV transmission and treatment is beyond the objective of this article.

However, it is instructive to show the extreme results according to the assumptions taken. In the most optimistic scenario, Granich and colleagues [27] concluded that with universal testing of the whole population at regular intervals, and immediate antiretroviral treatment for the infected patients, new infections could be virtually eliminated in South Africa within 10 years. In more pessimistic scenarios, behavioural disinhibition of infected people following the expansion of HAART could counteract any potential benefit and the epidemic would continue growing [28,29].

Lima and colleagues built a mathematical model to investigate different HAART coverage scenarios (50%, 60%, 75% and 100%) of those medically eligible to receive HAART under the 2008 IAS-USA guidelines in British Columbia, Canada [30]. A higher coverage scenario was calculated to cause a rapid initial fall in new infections followed by a gradual further decrease. Higher coverage caused initial excess costs, but the consequent prevention of new infections would save resources, so that HAART expansion would actually save money over a period of 50 years or so. With the new updated 2009 IAS-USA guidelines [9], the averted infections and the economic impact would be even more significant.

### Limitations of the "test-and-treat" approach

#### Impact of undiagnosed individuals on HIV transmission

Many new infections originate from persons who are HIV positive but undiagnosed. These patients are not receiving antiretroviral drugs and they can substantially contribute to the burden of the disease. "Test-and-treat" cannot achieve its full potential without expansion of testing. In addition, several researchers have found evidence of clusters of recently acquired infections in high-income countries [31,32], suggesting that a substantial proportion of transmission events originate from persons who are themselves recently infected. Depending on the screening test used, some of these persons may test falsely negative. "Test-and-treat" programmes must plan for this contingency, perhaps starting with two waves of testing a few months apart, and using contact tracing to find persons who were exposed to recent seroconverters.

#### Expanded testing

To identify most or all HIV-infected individuals in a population, testing must increase. However, acceptability of HIV testing is highly variable among countries, mostly related to the fear of stigma. This issue has been examined by a random sample of persons approached and offered HIV testing in 12 countries, including nine countries in sub-Saharan Africa (representing 18% of the epidemic in Africa). Results showed striking differences: whereas more than 60% agreed to be tested and received the results of HIV tests in the Dominican Republic and Swaziland, this proportion fell to 10% in Democratic Republic of Congo, and to 7% in Ethiopia [33].

#### From testing to retention in care

**Figure 3:**
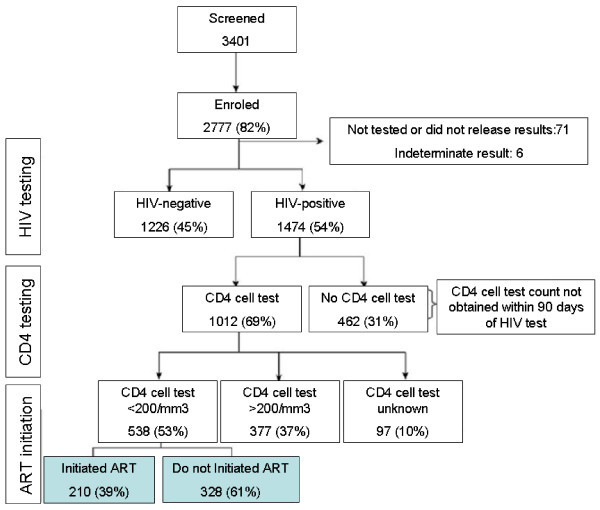
**Attrition in a HAART initiation study**. Study enrolment, HIV test results, CD4 cell count results and HAART initiation in two clinics of Durban, South Africa. Adapted from Bassett *et al*, *AIDS *2010, 24(Suppl 1):S37-S44 (with permission).

Patients, once they have been identified as HIV infected, do not all have CD4 cell count tests performed. If they have CD4 cell count tests, not all eligible patients will start HAART; HAART will not be effective in all who have this treatment; and some patients with effective treatment will discontinue. This cascade is convincingly presented in the real-life description of an urban clinic in Durban, South Africa [34]. After identifying 3400 people living with HIV, 82% were enrolled, of whom 69% had CD4 cell tests, but only 39% of eligible patients were started on HAART (Figure 3). Bendavid and colleagues concluded that increasing linkages to care and preventing loss to follow up could provide nearly twice the benefits of universal testing and treatment alone [35]. If patients are not retained in care after HAART initiation, the "test-and-treat" strategy will have little impact on prevention.

#### Impact of HAART on risk behaviour

HAART improves health and well-being and has changed the perception of HIV infection [36], considered now to be a manageable chronic disease. This may lead some patients to abandon safe sex, a phenomenon called risk compensation [37,38]. Risk compensation has been considered important enough in some mathematical models to counterbalance any positive impact of HAART expansion on transmission [28,29]. However, while the probability of transmission from a person on HAART with undetectable viremia cannot be quantified, it is probably low; indeed, only a single case report has so far been published [39]. Thus, even if risk compensation did occur, its effect on HIV transmission would be blunted by effective HAART in adherent patients. In addition, it should be noted that no risk compensation has been observed so far in the trials evaluating circumcision and topical microbicides [40-43].

#### Access to HAART and costs

Expansion of HAART will increase costs in the short run, i.e., during the first five to 20 years of the expansion. Later on, if expansion indeed reduces transmission, there are potential savings. The balance between investment and return depends on assumptions regarding costs of expanded testing, monitoring, drugs, uptake and efficacy of treatment, and changes in risk behaviour (among others).

Opinions regarding these assumptions vary widely. For example, Johnston and colleagues have estimated that over 30 years, a HAART expansion scenario from 50% to 75% of clinically eligible individuals would be associated with a net benefit of US$900 million in British Columbia, Canada [44]. On the other hand, in the wake of the economic recession of 2008, global budgets for antiretroviral treatment have stagnated. Patients who are already on treatment will need drugs for many years, while others, with deepening immune deficiency, will add to the numbers of those who need treatment. It will be a challenge to fulfil that need during the next 10 years, and perhaps unrealistic to plan for expansion of treatment in the hope of a hypothetical benefit in a generation or two.

### Perspectives: the research agenda

HPTN052 is a randomized study to evaluate HAART in preventing sexual transmission in serodiscordant couples. This study has been fully recruited since May 2010, and it includes 1750 couples where the infected partners have CD4 counts of 350 to 550 cells/mm^3^. In the intervention group, HAART is started at enrolment, while in the control group, HAART is started according to the local indication in the country where the study takes place. The endpoints of the study are the number of transmission events. Planned follow up will last at least five years; thus the first results are expected in 2015.

This is a well-designed, pioneering study, but in the general population, the methodology used in HPTN052 is not applicable. It would entail: (1) testing a whole population; (2) randomizing all HIV-positive people to either immediate or delayed treatment; and (3) following all sexual partners to check whether infection occurs. As stated in the section of serodiscordant couples, monogamy cannot be assumed in these types of studies. A so-called "cluster randomized trial" is a more realistic proposition. In such a trial, the unit of randomization is not the individual, but a community of individuals, for example, a town.

A study of this type is planned in South Africa; it will include approximately 30 clusters. An attempt will be made to screen all inhabitants of all clusters for HIV. In the intervention clusters, all who screen HIV positive will be treated, while in the control clusters, HAART will be administered only to patients with treatment indications according to local guidelines. The primary endpoint will be the number of incident HIV infections as measured by repetitive six-monthly screening. Several secondary endpoints would be considered, such as acceptability and the results of widespread testing, behavioural modifications, cost and cost effectiveness, and morbidity and mortality in the HIV-positive population.

A study of this type represents a priority in AIDS prevention research for the years to come, but it faces formidable obstacles. To start with, universal testing must be done in a way that avoids pressure, stigma and discrimination; this is not an easy undertaking. Success depends on maintaining a meaningful difference between intervention and control clusters. But in intervention clusters, not all will be screened, and of those found to be seropositive, not all will be treated. Of those who are treated, not all will have effective treatment, and of those with effective treatment, some will drop out and stop taking the medication. Meanwhile, in the control clusters, some are already on treatment, and many others will start during the trial as immune deficiency deepens and indications for treatment expand.

Other prevention methods, such as condoms and abstinence today and perhaps circumcision and microbicides tomorrow, will be used in both types of clusters. All these factors are expected to obscure the differences between intervention and control clusters at the risk of making the study inconclusive.

Such a trial has been proposed to the National Association for AIDS Research in France, and has been partially funding at the time of this writing. If such a trial demonstrates a benefit, public health authorities will need to evaluate the potential advantage of local decrease in HIV transmission over the financial cost of this strategy [45].

## Conclusions

In conclusion, a growing body of circumstantial evidence suggests an important preventive role for HAART. While waiting for results of definitive trials, maximum efforts must be made to ensure that every patient with clinical indication is treated. In resource-poor settings, providing treatment for all patients with CD4 counts of less than 350 cells/mm^3 ^already represents a considerable challenge. Ensuring equitable access to testing, counselling and HAART in every country according to national guidelines would at least represent a first important step towards optimizing the prevention effect of antiretroviral drugs.

## Competing interests

BH and AC are involved in planning a cluster-randomized trial, called "TasP" (Treatment as Prevention), in South Africa, and funded by the French Agency for Research on AIDS and Hepatitis (ANRS). JA has no competing interests to declare.

## Authors' contributions

BH presented a plenary session of "HIV Treatment as Prevention" at the XVIII International AIDS Conference (AIDS 2010) in Vienna. With this presentation as the starting point, JA wrote the initial draft of the paper, which then underwent revision and final approval by all authors.
